# Prospect theory and body mass: characterizing psychological parameters for weight-related risk attitudes and weight-gain aversion

**DOI:** 10.3389/fpsyg.2015.00330

**Published:** 2015-03-24

**Authors:** Seung-Lark Lim, Amanda S. Bruce

**Affiliations:** ^1^Department of Psychology, University of Missouri – Kansas CityKansas City, MO, USA; ^2^Department of Pediatrics and Center for Children's Healthy Lifestyles and Nutrition, University of Kansas Medical Center and Children's Mercy HospitalKansas City, KS, USA

**Keywords:** decision-making, prospect theory, loss aversion, risk seeking, obesity

## Abstract

We developed a novel decision-making paradigm that allows us to apply prospect theory in behavioral economics to body mass. 67 healthy young adults completed self-report measures and two decision-making tasks for weight-loss, as well as for monetary rewards. We estimated risk-related preference and loss aversion parameters for each individual, separately for weight-loss and monetary rewards choice data. Risk-seeking tendency for weight-loss was positively correlated with body mass index in individuals who desired to lose body weight, whereas the risk-seeking for momentary rewards was not. Risk-seeking for weight-loss was correlated to excessive body shape preoccupations, while aversion to weight-gain was correlated with self-reports of behavioral involvement for successful weight-loss. We demonstrated that prospect theory can be useful in explaining the decision-making process related to body mass. Applying prospect theory is expected to advance our understanding of decision-making mechanisms in obesity, which might prove helpful for improving healthy choices.

## Introduction

Prospect theory is a behavioral economic theory developed by Daniel Kahneman. His work with Amos Tversky, namely prospect theory, provides a way to describe how people make decisions based on psychological valuation of potential gains and losses (Kahneman and Tversky, 1979). Prospect theory provides an augmentation to neoclassical economic utility models, and is a descriptive approach to human behavior that explains how people *actually* make decisions rather address how people theoretically *should* make decisions. In other words, prospect theory describes or characterizes real-life choices, which are often not optimal, rather than explaining rational choices. Prospect theory studies typically employed monetary choices that include positive and negative outcomes (e.g., winning or losing money). Interestingly, the majority of people in the United States view weight-loss as a positive outcome and weight-gain as a negative outcome (Robison et al., 1993). Like many human behaviors, our eating behaviors are often “less than optimal.” For example, though we know spinach is healthy and have a desire to have a healthy body weight, we often choose to eat French fries instead. Furthermore, some people opt for pharmacological or surgical treatments to control body weight that may cause negative side effects (e.g., yo-yo effect) instead of changing their eating behavior. Observations of eating and body-weight management behaviors may introduce us the opportunity to apply prospect theory to better understand basic decision-making mechanisms in obesity.

**Figure 1:**
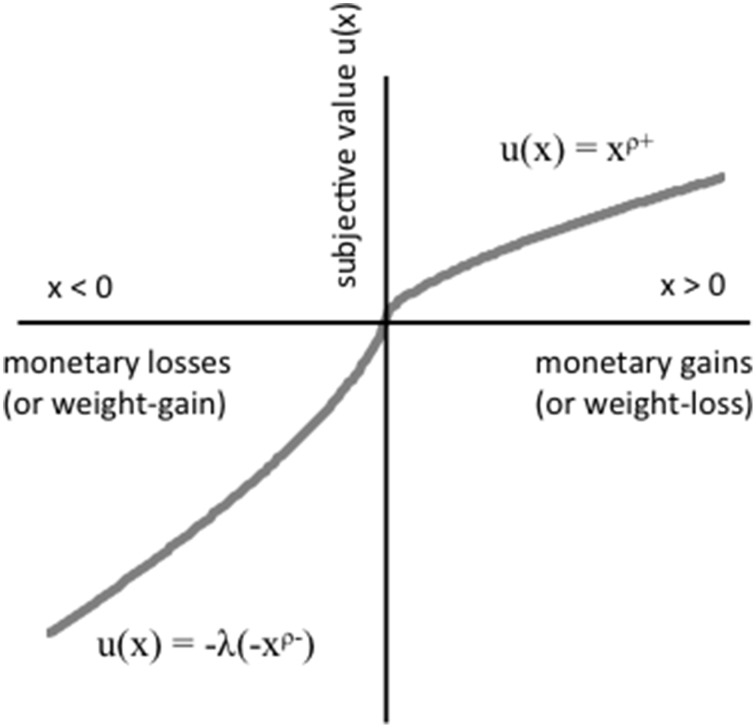
**A hypothetical value function based on prospect theory**.

Prospect theory includes the concept of risk preference (also referred to as risk-seeking or risk-aversion) and loss aversion, which is illustrated in Figure 1. Risk preference refers to the extent to which people are comfortable with *probabilistic* gains or losses. Commonly, in prospect theory, these gains and losses are operationalized using monetary values. There are wide individual differences in risk preference, supported by a wealth of psychological research (Tversky and Kahneman, 1992; Tversky and Fox, 1995). Prospect theory can explain why risk-seeking individuals prefer an uncertain option to a certain option (e.g., 50% chance of $100 rather than 100% chance of $50), while risk-averse individuals are reluctant to accept an uncertain option even when it has an equal or greater expected payoff than a certain option (e.g., a gamble that has 50% chance of winning $100 and 50% chance of losing $50). Risk preference also follows a developmental trajectory with adolescents typically more risk-seeking than young and middle adults (Steinberg, 2004). Risk-seeking behavior is thought to be related to impulsivity and diminished executive control (Kelley et al., 2004; Gladwin et al., 2011).

Recently, a growing body of literature has demonstrated obese individuals show greater impulsivity (Braet et al., 2007; Epstein et al., 2010; Jarmolowicz et al., 2014) as well as greater reward sensitivity when compared to healthy weight individuals (Nederkoorn et al., 2006; Carnell et al., 2012). For example, obese individuals were more sensitive to food rewards and revealed the inability to control eating behaviors (Nederkoorn et al., 2006). Similarly, a functional neuroimaging study showed that elevated body weight correlated with impulsivity measured by behavioral response inhibition task that accompanied with decreased neural responses in inhibitory control regions and increased neural responses in food-related reward regions (Batterink et al., 2010). Also, previous research has documented excessive temporal discounting (increased delay discounting rates) in individuals carrying excess weight individuals (Weller et al., 2008; Epstein et al., 2010; Bickel et al., 2014; Jarmolowicz et al., 2014). That is, obese individuals are more likely to choose a smaller reward *sooner* than a larger reward *later.*

Overall, mounting evidence suggests that executive functions might be altered in individuals with excess body weight (Verdejo-Garcia et al., 2010; Reinert et al., 2013; Liang et al., 2014). Thus, it is possible obese individuals tend to be higher in risk-seeking than healthy weight individuals in probabilistic choice situations, though few studies have examined this; one study reported that most of patients who seeking weight-loss surgery showed willingness to accept risk of dying to undergo surgery (Wee et al., 2013). In particular, regarding probabilistic choices, it is not fully understood whether obese individuals would be more likely to generally engage in risky behaviors across domains (including monetary choices), or whether they would be more likely to engage in risky behaviors specific to weight-related decisions.

Loss aversion refers to the idea that people would much rather *avoid a loss* than they would gain a reward, and there is substantial theoretical and empirical support for the construct (Camerer, 2005). Loss aversion is represented by a steeper slope in the region of losses (*x* < 0) than in the region of gains (*x* > 0) (see Figure 1). In general, we hate losing much more than we like winning. Using a monetary example, a chance to lose $100 (psychological loss/pain) can be *twice* as impactful on our behavior as a chance to win $100 (psychological gain/pleasure). There are also individual differences in loss aversion, with some individuals particularly averse to situations that include possible losses (Tversky and Kahneman, 1991; De Martino et al., 2010).

By using probabilistic choice sets that include both gains and losses, prospect theory can parameterize individual differences in loss aversion and risk preference. To our knowledge, no studies have examined whether risk preference and loss aversion differ based on body mass. In behavioral economics research, risk preference and loss aversion are most commonly examined using objects that have monetary values. It may be worthwhile, however, to also investigate how the *mode* of reward affects behavioral decisions. In fact, a paper examining behavioral economics of childhood overweight and obesity called for a more thorough examination of constructs of prospect theory (Ehmke et al., 2008). If people perceive *weight-gain* as a “psychological loss” and *weight-loss* as a “psychological gain” (Robison et al., 1993), it would be possible to investigate psychological parameters for weight-related risk preference and weigh-gain aversion. If applying the concept of loss aversion to body mass, we would assume people would be much more sensitive to gaining weight (psychological loss/pain) than losing weight (psychological gain/pleasure) of the same or larger magnitude. We will refer to this as “weight-gain aversion.”

Our study aimed to develop a novel decision-making paradigm that allows us to apply prospect theory in behavioral economics to body mass. Critically, the subjective value of weight (gaining weight or losing weight) may not correspond to the absolute weight value. We believe that understanding mechanisms of psychological valuation of body weight would provide important information for obesity research as well as successful weight management. The current study is designed to further our understanding of the relationship between body mass index (BMI) and psychological constructs within prospect theory. We hypothesized that the psychological phenomenon of risk preference and loss aversion would be observed related to body weight for individuals who have a desire to lose their weight, and the individual difference in prospect model parameters from weight-loss choices would be specifically correlated to body mass and obesity-related psychological variables.

## Methods and procedures

### Participants

Sixty-seven healthy college students with a mean age of 23.0 (*SD* = 6.0 years; 13 males) were recruited through Psych Pool online research participant recruitment system at the University of Missouri–Kansas City (UMKC). Participants received course credits for participating in the experiment. The study protocol was reviewed and approved by UMKC's Institutional Review Board. Prior to the experiment, participants provided informed consent and completed a demographics questionnaire including two questions that asked whether they were at desired weight and, if not, what percentage of body weight they want to lose or gain with a rating scale (−30,−20,−10,−5%, 0, 5, 10, 20, 30%).

Participants also completed Weight Locus of Control Scale (WLOC) (Saltzer, 1982), Body Shape Questionnaire (BSQ) (Cooper et al., 1987), and Eating Behavior Inventory (EBI) (O'neil et al., 1979). The WLOC scale (Saltzer, 1982) measures the expectancy that one can control weight changes in a 6-point Likert scale. The WLOC includes two internal control items and two external control items. The Cronbach's alpha coefficient in this study was 0.46. The BSQ (Cooper et al., 1987) includes 34 items that measure weight and body shape preoccupations caused by being fat. It has been used to assess eating disorder pathology in clinical settings (Anderson et al., 2004). The Cronbach's alpha of the BSQ in this study was 0.98. The EBI (O'neil et al., 1979) is a self-report instrument that assesses frequencies of behaviors that are associated with weight loss and weight management. The EBI is known to be consistently sensitive to successful behavioral weight management interventions (O'neil and Rieder, 2005). The Cronbach's alpha of the EBI in this study was 0.71.

Body weight and height were measured to calculate Body Mass Index (BMI) (kg/m^2^). Among the 67 participants (42 females, 13 males; 15% freshman, 24% sophomore, 28% junior, 33% senior; 55% Caucasian, 3% Hispanic, 19% African American, 17% Asian, 6% Others), 27 participants (40%) answered that they were at a desired weight, 35 participants (53%) answered that they were not at a desired weight and wanted to lose weight, and five participants (7%) answered that they were not at desired weight and wanted to gain weight. The latter five participants, who may approach decisions about losing weight differently than individuals in other two groups, were excluded from further data analyses.

Among 35 participants who expressed that they were not at their desired weight and wished to lose their body weight (Unsatisfactory Group), 13 (37%) were in the normal weight range (BMI: 18.5 ~ 25), 14 (40%) were in the overweight range (BMI: 25 ~ 30), and 8 (23%) were in obese range (BMI: 30 or higher). Among 27 participants who expressed that they were at desired weight and reported no desire to change their body weight (Satisfactory Group), 2 (8%) were in underweight status (BMI: below 18.5), 19 (70%) were in normal weight status, and 6 (22%) were in overweight status. The unsatisfactory and satisfactory groups showed a significant difference in BMI scores (*t* = 5.54, η^2^ = 0.34, *p* < 0.001; *M*_unsatisfactory_ = 27.71, *SE* = 0.75; *M*_satisfactory_ = 22.23, *SE* = 0.57), but there were no significant difference between gender (χ^2^ = 0.20, *df* = 1, *p* = 0.89), college year (χ^2^ = 0.80, *df* = 3, *p* = 0.85), or ethnicity (χ^2^ = 2.73, *df* = 4, *p* = 0.60).

### Experimental paradigm

**Figure 2:**
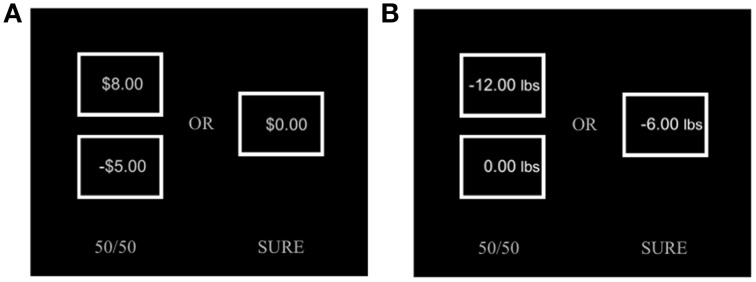
**Sample displays of choice sets [(A) monetary choice, (B) weight-loss choice] are shown**. The top and bottom boxes on the left represent the possible monetary gain (or weight-loss) and monetary loss (or weight-gain) for an 50%/50% uncertain option, the box on the right represent an 100% sure option. Participants were required to make forced choices between the two hypothetical options.

Participants completed two probabilistic decision-making tasks for monetary rewards and weight-loss based on prospect theory. Each task contained 140 forced binary choice trials. As shown in Figure 2, each choice trial included two choice options between a risky gamble (50% chance of winning) and a guaranteed amount of rewards (100% chance of winning). The probability of gambles was fixed to *P* = 0.50. All choices were hypothetical, but participants were encouraged to consider their choices that could be implemented as real outcomes (i.e., winning money or losing body-weight as shown). To characterizing psychological parameters of the prospect theory model–(a) attitude toward chance (risk reference parameter for gain and loss, ρ^+^ and ρ^−^) and (b) subjective weighting of losses over gains (loss aversion parameter, λ), the values of gains (monetary rewards or body-weight loss) and losses (monetary losses or body-weight gain) were parametrically varied across choice trials. Monetary choice sets were taken from Sokol-Hessner and colleagues' study (Sokol-Hessner et al., 2009). The exact same values from the monetary reward choice set were used for the body-weight choice set, but shown in a unit of body weight (pounds) instead of the U.S. dollar ($). For example, $10 win in monetary choices corresponded to 10 lbs. body-weight loss in body-weight choices. Similarly, $5 loss in monetary choices corresponded to 5 lbs. body-weight gain in body-weight choices. This exploratory conversion was employed to achieve a realistic range of weight gain and loss choices (24 lbs weight loss ~ 30 lbs weight gain; See Appendix for a full list of choices). Note that our conversion does not necessarily equate utilities of gambles from two different domains, nor make them directly comparable, which were not our research goals. Among 140 choices for each set, 120 were forced-choices between mixed-valence gambles (50% positive + 50% negative) and guaranteed (100%) amounts of zero. The remaining 20 choices did not include negative outcomes, in which participants made forced-choices between gain only gambles (50% positive + 50% zero) and guaranteed (100%) amounts of positive outcomes. Choices were presented in random order. Participants were asked to indicate their choices by pressing one of two keyboard buttons (number “1” for the left option; number “0” for the right option). A choice set was displayed on monitor screen until a button press. After a button press, only the chosen option remained on the screen for an additional 0.5 s. Every trial was separated by an inter-trial-interval of a 1.5 s fixation cross. The experimental schedule of stimulus presentation and behavioral data acquisition were programmed using Presentation software (Neurobehavioral System, Berkeley, CA).

### Prospect theory model parameter estimation

Each individual participant's prospect theory model parameters were estimated through a nonlinear stochastic choice model following approaches described in the previous study (Sokol-Hessner et al., 2009). From Kahneman and Tversky's prospect theory (Kahneman and Tversky, 1979), the participant's utility (value) functions were represented as two parts of a power function by the sign of *x* values (gains: *x* > 0; losses: *x* < 0) as shown below.

$$u(x)=\{\begin{array}{cc} x^{\rho^{+}} & ifx\geq 0\\ -\lambda \left(-x^{\rho^{-}}\right) & ifx<0 \end{array}$$

We estimated utility functions separately for monetary rewards and body-weight loss choices. For body-weight loss choices, the weight-loss was conceptualized as a *gain* domain that implies potential motivational incentives, while the weight-gain was conceptualized as a *loss* domain. Note that we excluded those five participants who expressed a desire to gain body weight as described before.

This prospect theory model included three parameters–(1) the risk preference parameter for a gain domain (monetary gains or weight loss), ρ^+^, (2) the risk preference parameter for a loss domain (monetary losses or weight gain), ρ^−^, and (3) the loss aversion parameter, λ. We selected two-risk preference parameters model rather than one-risk preference parameter model (i.e., a single ρ for gain and loss domains), because it was plausible that participants might show different risk-related attitudes for gain and loss domains, particularly for weight gain and weight loss. The risk preference parameter (ρ) represents diminishing sensitivity (a discounting rate) to changes in value for the increase in absolute value. A ρ-value larger than 1 indicates the risk-seeking attitude—a preference for an uncertain option over a certain option. On the other hand, a ρ-value smaller than one indicates the risk-averse attitude—the reluctance to accept an uncertain option that may have an equal or higher expected payoff over a certain option. Prospect theory predicts that risk-seeking and risk-averse individuals would show different behaviors for a binary choice between a risky gamble (50% for $20 + 50% for $0) and guaranteed money of $10, although both options have the same expected values of $10. If ρ^+^_money_ = 0.8, the risky gamble and the guaranteed money would have subjective values of $5.49 (=20^0.8^ × 0.5) and $6.31 (=10^0.8^), respectively. In this scenario, a risk-averse person of ρ^+^_money_ = 0.8 would choose the guaranteed $10 option. Similarly, by applying the prospect theory model to weight-loss choices, we can predict that weight-loss risk-seeking individuals would likely to choose the risky weight-loss option over the guaranteed weight-loss option, while risk-averse individuals would likely to choose the opposite. For another example, a choice between the risky weight-loss (50% for 20 lbs. + 50% for 0 lbs.) and the guaranteed weight-loss of 10 lbs., if ρ^+^_weight_ = 1.2, a risk-seeking person (who has a desire to lose weight) will choose the risky weight-loss gamble that has a subjective value of 18.21 lbs. (= 20^1.2^ × 0.5) rather than the sure weight-loss that has a subjective value of 15.85 lbs. (= 10^1.2^). Thus, smaller ρ represents stronger risk-averse attitude, while larger ρ represents a stronger risk-seeking attitude. A ρ-value of one indicates risk neutrality or indifference. In the prospect theory model, the loss aversion parameter (λ) represents relative weighting of losses (e.g., losing money) over gains (e.g., earning money). As shown in the equation above, the value function of loss domain (*x* < 0) is calculated by multiplying this loss aversion parameter. For our body-weight loss choices, the weight-gain was treated as a negative consequence. Thus, the aversion parameter for weight gain (λ_weight_) represents relative multiplicative weighting of weight-gain over weight-loss. A value of λ_weight_ > 1 represents a tendency to overemphasize weight-gain relative to weight-loss in subjective valuations of weight changes.

Model parameter estimations were performed following Sokol-Hessner et al.'s procedure (Sokol-Hessner et al., 2009). For each individual, the probability to accept 50% gambles (*P_gamble_*) can be described as below. The logit sensitivity or slope parameter (μ), that represents the sensitivity of choice probability to the subjective utility differences or the amounts of inconsistency (randomness) over choices were estimated through a softmax function.

$$P_{gamble}={\left(1+\exp\{-\mu (u_{gamble}-u_{guranteed})\}\right)}^{-1}$$

Separately for monetary choices and weight-loss choices, we fitted the data using maximum likelihood, with the log likelihood function implemented with the MATLAB software (MathWorks, Natick, MA) to estimate prospect theory model parameters.

$$\sum_{i\;=\;1}^{140}y_{i}\log(P_{gamble})+(1-y_{i})\log(1-P_{gamble})$$

Here, *y_i_* represents the choice of participant in trial *i* (1 for gamble choices, 0 for guaranteed alternative choices). See Sokol-Hessner et al.'s for additional details on estimation methods (Sokol-Hessner et al., 2009).

### Statistical data analysis

After individually estimating prospect theory model parameters, we conducted a series of independent *t*-tests to compare the estimated model parameters between the unsatisfactory (*n* = 35) and satisfactory groups (*n* = 27). Parameter estimations and comparisons were performed separately for weight-loss and monetary choices. Next, we performed exploratory correlations and multiple linear regression analyses with the prospect theory model parameters, BMI, and psychosocial variables included in our study.

## Results

We speculated that psychological values (or “utilities”) of weight-gain and weight-loss might differ according to individuals' attitudes toward their current body weight and whether they desire to lose body weight or not. Therefore, we have summarized statistical results separately for unsatisfactory (i.e., desire to lose body weight) and satisfactory (i.e., no desire to lose or gain body weight) groups for data analyses. For each participant, we fitted the prospect theory model parameters separately for weight-loss choices and monetary choices.

### Prospect theory model parameters for weight-loss and monetary choices

A prospect theory model parameter summary for weight-loss choices and monetary choices is shown in Table 1. The unsatisfactory group's mean model parameters for weight-loss risk preference (ρ^+^_weight_), weight-gain risk preference (ρ^−^_weight_), and weight-gain aversion (λ_weight_), which were estimated from hypothetical weight-loss choices, were 0.88, 1.04, and 2.09. The satisfactory group's mean parameters for ρ^+^_weight_, ρ^−^_weight_, and λ_weight_ were 0.43, 0.93, and 1.93. The unsatisfactory and satisfactory group showed a significant difference in the weight-loss risk preference parameter (ρ^+^_weight_; *t* = 4.82, η^2^ = 0.28, *p* < 0.001), while they did not show a significant difference in the weight-gain risk preference parameter (ρ^−^_weight_; *t* = 0.75, η^2^ = 0.01, *p* = 0.46). Interestingly, both unsatisfactory and satisfactory groups showed weight-gain aversion (λ_weight_ > 1; *t* = 3.71, η^2^ = 0.29, *p* < 0.01; *t* = 2.94, η^2^ = 0.25, *p* < 0.01) that was not statistically different each other (λ_weight_; *t* = 0.37, η^2^ = 0.00, *p* = 0.71), suggesting that even for participants at the desired body weight, gaining body weight has relatively larger psychological impact compared to losing body weight. On the other hand, as expected, the unsatisfactory and satisfactory groups did not show any significant difference for prospect model parameters of monetary choices (all *p* > 0.05).

**Table 1:** **Prospect theory model parameters for unsatisfactory and satisfactory groups**.

**Parameter**	**Unsatisfactory group (*n* = 35)**	**Satisfactory group (*n* = 27)**	***t***	**η^**2**^**	***p***
ρ^+^_weight_	0.88 (0.06)	0.43 (.07)	4.82	0.28	<0.001
ρ^−^_weight_	1.04 (0.09)	0.93 (0.12)	0.75	0.01	0.46
λ_weight_	2.09 (0.30)	1.93 (0.32)	0.37	0.00	0.71
ρ^+^_monetary_	0.99 (0.05)	0.88 (0.07)	1.34	0.03	0.18
ρ^−^_monetary_	0.86 (0.06)	0.85 (0.08)	0.13	0.00	0.89
λ_monetary_	2.30 (0.28)	2.65 (0.36)	−0.78	0.01	0.44

Means and standard errors are shown. Higher ρ scores denote risk-seeking tendency, while higher λ scores denote monetary loss aversion or weight-gain aversion.

To explore how prospect theory model parameters for weight-loss choices and monetary choices, we computed pairwise correlations between two modalities. In the unsatisfactory group, all three parameters (ρ^+^, ρ^−^, and λ) showed significant positive correlations (*r* = 0.37, *p* < 0.05; *r* = 0.50, *p* < 0.01; *r* = 0.47, *p* < 0.01). However, in the satisfactory group, only ρ^−^ parameter revealed a significant correlation, (*r* = 0.44, *p* < 0.05), while ρ^+^ and λ parameters did not (*r* = 0.17, *p* = 0.39; *r* = 0.24, *p* = 0.23). Our result suggests that weight-related risk preference and weight-gain aversion attitudes share common variance with risk preference and loss aversion attitudes estimated from traditional monetary choices, and demonstrates convergent validity in the individuals who want to lose weight.

### Body mass and prospect theory model parameters

**Figure 3:**
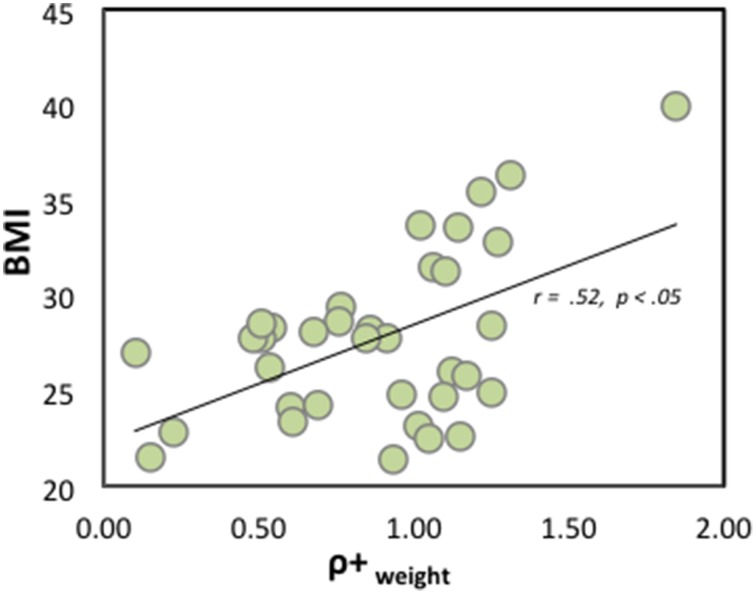
**Scatter plot between weight-loss risk preference parameters and BMI scores in satisfactory group**. ρ^+^_weight_ = 1 represents risk neutrality. ρ^+^_weight_ > 1 represents risk-seeking, whereas ρ^+^_weight_ < 1 represents risk-aversion. Note significant correlation even after removing an individual with BMI of 40.

To investigate the relationship between prospect theory model parameters and obesity, we performed correlational analyses separately for the unsatisfactory and satisfactory groups. As illustrated in Figure 3, BMI scores were positively correlated with weight-loss risk preference parameter, ρ^+^_weight_ (*r* = 0.52, *p* < 0.01) in the unsatisfactory group. In other words, obese individuals (who want to lose weight) tend to choose risky (uncertain) weight-loss options rather than safe (certain) weight-loss options (e.g., taking 50% chance of 12 lbs. weight-loss rather than 100% chance of 6 lbs. weight-loss). Interestingly, the risk preference parameter of monetary gains (ρ^+^_monetary_) did *not* show a significant correlation (*r* = −0.06, *p* = 0.75), suggesting the specificity of association of obesity with a risk-seeking tendency for “weight-loss,” which cannot be measured via other domains of incentives such as monetary rewards. Our weight-loss choices employed the absolute amount of potential weight loss or gain. However, subjective valuation for the *absolute* amount of weight loss could be different across participants, and it might affect the first-order correlation between BMI scores and weight-loss risk preference parameter (ρ^+^_weight_). To rule out this possibility, we performed additional partial correlation analysis with a rating of the percentage of weight loss or gain to want to achieve. Even after controlling for the percentage of weight change ratings, BMI showed a significant positive relationship with a risk-seeking tendency for weight-loss (*r_partial_* = 0.38, *p* < 0.05) in the unsatisfactory group. The other prospect model parameters did not show a significant correlation with BMI scores (all *p* > 0.05; Table 2). Also, not surprisingly, no significant correlation between BMI scores and risk preference parameters existed in the satisfactory group.

**Table 2:** **Pearson correlations between prospect theory risk-seeking and weight-loss aversion parameters and self-report scales in unsatisfactory and satisfactory groups**.

**Parameter**	**BMI**	**WLOC^a^**	**BSQ^b^**	**EBI^c^**
**UNSATISFACTORY GROUP (*n* = 35)**				
ρ^+^_weight_	0.52 (p = 0.001)	−0.22 (p = 0.20)	0.37 (p = 0.03)	0.14 (p = 0.44)
ρ^−^_weight_	0.18 (p = 0.29)	0.02 (p = 0.93)	0.30 (p = 0.08)	0.06 (p = 0.72)
λ_weight_^b^	0.12 (p = 0.51)	0.00 (p = 0.99)	−0.02 (p = 0.93)	0.39 (p = 0.02)
**SATISFACTORY GROUP (*n* = 27)**				
ρ^+^_weight_	0.11 (p =0.59)	−0.03 (p = 0.89)	0.45 (p = 0.02)	−0.01 (p = 0.97)
ρ^−^_weight_	0.02 (p = 0.94)	0.03 (p = 0.88)	0.10 (p = 0.63)	−0.11 (p = 0.58)
λ_weight_^b^	−0.02 (p = 0.94)	−0.16 (p = 0.42)	0.09 (p = 0.66)	0.11 (p = 0.62)

Correlation coefficients and p values are shown.

a Weight Locus of Control Scale; Higher scores denote perceived external locus of control of weight-loss.

b Body Shape Questionnaire; Higher scores denote more body shape preoccupations.

c Eating Behavior Inventory; Higher scores denote behaviors linked with weight-loss success.

### Obesity-related psychological variables and prospect theory model parameters

Next, to investigate the relationship between prospect model parameters and obesity-related psychological variables, we performed correlational analyses with self-report scales (Table 2). The ρ^+^_weight_ parameter was positively correlated with BSQ (*r* = 0.37, *p* < 0.05; *r* = 0.45, *p* < 0.05) in both unsatisfactory and satisfactory groups, showing that risky weight-loss choices are associated with body shape preoccupations. Additionally, we performed a multiple regression analysis on ρ^+^_weight_ parameter with all three psychological variables as well as with BMI in the unsatisfactory group. The BMI and BSQ significantly predicted ρ^+^_weight_ parameter even after controlling for other variables (*t* = 2.12, *r*^2^_*part*_ = 0.09, *p* < 0.05; *t* = 2.39, *r*^2^_*part*_ = 0.12, *p* < 0.05). On the other hand, the λ_weight_ parameter revealed a significant positive correlation with EBI (*r* = 0.38, *p* < 0.05). This result suggests that weight-gain aversion estimated via prospect theory is closely associated with active behavioral involvement for successful weight-loss. For completeness, we checked additional correlations between the estimated parameters from monetary choices and self-report scales. As expected, none of correlations was significant in both groups, confirming the specificity of prospect theory parameters for weight-loss.

## Discussion

This study demonstrates that prospect theory, widely utilized to understand human economic decisions, can also be applied to other domains. It can provide novel and valuable information to understand underlying decision-making mechanisms related to obesity. Daily decisions are critical determinants for a healthy life. Foods we choose to eat (energy intake) and if and how much we exercise (energy expenditure) directly affect our weight status. In prospect theory, the value of outcome does not necessarily correspond to the objective, monetary value. Our results demonstrate that neither does the objective value of weight correspond to the absolute weight value. The psychological parameters estimated from weight-loss choices displayed similar characteristics to the monetary choices in individuals who desired to lose weight, showing significant correlations for risk preference and loss aversion parameters. Furthermore, we confirmed and better characterized weight-gain aversion similar to loss aversion for monetary values in both unsatisfactory and satisfactory groups. We have referred to this concept as “weight-gain aversion.” As expected, the threat of weight-gain looms larger than an equivalent opportunity of weight-loss. In other words, participants detest weight gain about twice as much as they enjoy weight-loss. The relative ratio of weight-gain aversion in the unsatisfactory and satisfactory groups were 2.09 (*SE* = 0.30) and 1.93 (*SE* = 0.32), which were close to the typical parameter range of 1.5 ~ 2.5 from previous studies with monetary rewards (Tversky and Kahneman, 1992; Bateman et al., 2005; Tom et al., 2007; Sokol-Hessner et al., 2009, 2013).

Although this single study cannot determine *why* people detest weight-gain more than they like weight-loss, a positive correlation between two loss aversion model parameters in the unsatisfactory group suggest that loss aversion for monetary loss and weight gain may share common underlying mechanisms. It has been suggested that hypersensitive to losses is driven by or mediated by negative emotions such as fear or anxiety (Camerer, 2005). Thus, weight-gain aversion observed in our study also might be driven by fear of being obese. A neuroimaging study showed that individual indifferences in loss aversion in monetary choices correlate with brain activities in the ventral striatum and prefrontal cortex (Tom et al., 2007). Thus, it would be interesting to explore underlying neurobiological mechanisms of weight gain aversion in future studies. We also observed the unique role for prospect theory's weight-gain aversion in relation to psychological variables. Overall, our results of weight-loss choices suggests that prospect theory can successfully describe how an individual subjectively perceives weight-gain and weight-loss and how s/he processes uncertainty related to weight changes.

In our study, individuals who want to lose weight showed a increased tendency for risky choices for weight-loss, compared to individuals who are satisfied with their current weight. Also, the risk-seeking parameter for weight-loss was positively correlated with body mass in the individuals who had a desire to lose their body weight, while the risk-seeking parameter for monetary rewards was not. A positive correlation between the risk-seeking for weight-loss and body mass was observed even after statistically controlling for the percentage amount of weight-loss wishes, suggesting that the amounts of desired weight loss might be not a critical factor that determines the relationship between risk-seeking for weight-loss and the body mass. Considering the previous research on the impulsivity and reward sensitivity in obesity (Nederkoorn et al., 2006; Braet et al., 2007; Epstein et al., 2010; Carnell et al., 2012; Jarmolowicz et al., 2014), our finding of the risk-seeking tendency for weight-loss is particularly interesting. It is plausible that the hypersensitivity to weight-loss (which is psychologically rewarding) and impulsivity (poor cognitive control) might contribute to make individuals choose an uncertain option (50% probability for larger weigh-loss) rather than a sure option (100% probability of smaller wegith0-loss). Also, the risk-seeking choices might share overlapping executive control mechanisms that have been shown to be affected by carrying excess body weight (Verdejo-Garcia et al., 2010; Reinert et al., 2013; Liang et al., 2014).

The present study further elucidates relations between risk-seeking for weight-loss and other psychological variables. Interestingly, the risk-seeking for weight-loss was related to the BSQ scores in our study. The BSQ, measures body shape preoccupation or concern often typically observed in eating disorders (Cooper et al., 1987). In regression analyses, BMI and BSQ significantly predicted risk-seeking for weight-loss even after controlling for the effects of other variables. Our data suggests that increased concern with body shape as well as excessive body mass may be uniquely associated with risk-seeking behaviors for weight-loss. On the other hand, aversion to weight-gain itself was not correlated with BMI, but it was positively correlated with EBI that measures behavioral involvement for weight management. Higher scores on the EBI indicate greater adoption of behaviors that are linked with successful weight-loss (e.g., “I carefully watch the quantity of food I eat”). Previous studies demonstrated that EBI is a sensitive predictor for weight-loss (O'neil et al., 1979). Thus, weight-gain aversion may serve as a motivational factor for active weight management behaviors in daily life.

Utilizing weight-loss choices from prospect theory provides valuable information that more general prospect theory parameters based on monetary values fails to predict. That is, prospect theory may provide a unique, individually-tailored therapeutic perspective for decision-making to achieve a healthier energy balance. As demonstrated through our results, applying prospect theory in obesity enables us to describe exact *subjective weight-value function* for each individual, which cannot be achieved through other decision-making tasks that have been applied in obesity research (e.g., Iowa gambling task, temporal discounting tasks) (Best et al., 2012; Daniel et al., 2013; Verbeken et al., 2014). However, there are several caveats. Our first-of-a-kind study cannot yet explain how prospect model parameters could be meaningfully leveraged to change real eating and/or weight-management behaviors. Also, it is still uncertain whether weight-loss risk-seeking and weight-gain aversion are malleable or stable traits. Recent studies demonstrate cognitive and affective regulation strategies can change parameters of prospect theory for monetary choices (Thaler et al., 1997; Sokol-Hessner et al., 2009, 2013). Thus, our finding may be helpful for future research to apply prospect theory to change obesogenic decision-making mechanisms food and weight-management related behaviors. Self-control interventions that directly target risk-seeking decision-making might be effective in weight-management in obese individuals. As mentioned in our introduction, several recent studies showed overweight and obese individuals are more lkely to choose smaller, more immediate monetary rewards (Borghans and Golsteyn, 2006; Weller et al., 2008; Ikeda et al., 2010; Bickel et al., 2014; Jarmolowicz et al., 2014), suggesting steeper temporal discounting of future rewards. Future studies that explore whether temporal discounting future rewards is related to the risk-seeking tendency for weight-loss would be informative. We acknowledge that our study utilized a sample of college students and in order to generalize our findings, future studies with larger samples including both community and clinical populations will be required. A more complete understanding of the complex relations between body mass and weight-loss risk-seeking and weight-gain aversion could inform health behavior interventions. Changing the process of how we decide may ultimately help us make healthier decisions.

### Conflict of interest statement

The authors declare that the research was conducted in the absence of any commercial or financial relationships that could be construed as a potential conflict of interest.
