# Supplementary material for: Prospect theory and body mass: characterizing psychological parameters for weight-related risk attitudes and weight-gain aversion
Source: Front Psychol. 2015 Mar 24;6:330. doi: 10.3389/fpsyg.2015.00330 (PMC4371555; doi:10.3389/fpsyg.2015.00330)
Supplement: Supplementary file 1 [file Table1.PDF]

**Appendix.** Amounts of gain (monetary gain $, weight loss lbs) and loss (monetary loss $, weight gain lbs) in the choice set (total 140 choices)

| | Gamble | | Ceartain |
|---|---|---|---|
| | Gain | Loss | Gain |
| 1 | 2 | 0.50 | 0 |
| 2 | 2 | 0.75 | 0 |
| 3 | 2 | 1.00 | 0 |
| 4 | 2 | 1.25 | 0 |
| 5 | 2 | 1.50 | 0 |
| 6 | 2 | 1.75 | 0 |
| 7 | 2 | 2.00 | 0 |
| 8 | 2 | 2.25 | 0 |
| 9 | 2 | 2.50 | 0 |
| 10 | 2 | 2.75 | 0 |
| 11 | 2 | 3.00 | 0 |
| 12 | 2 | 3.25 | 0 |
| 13 | 2 | 3.50 | 0 |
| 14 | 2 | 3.75 | 0 |
| 15 | 2 | 4.00 | 0 |
| 16 | 4 | 1.00 | 0 |
| 17 | 4 | 1.50 | 0 |
| 18 | 4 | 2.00 | 0 |
| 19 | 4 | 2.50 | 0 |
| 20 | 4 | 3.00 | 0 |
| 21 | 4 | 3.50 | 0 |
| 22 | 4 | 4.00 | 0 |
| 23 | 4 | 4.50 | 0 |
| 24 | 4 | 5.00 | 0 |
| 25 | 4 | 5.50 | 0 |
| 26 | 4 | 6.00 | 0 |
| 27 | 4 | 6.50 | 0 |
| 28 | 4 | 7.00 | 0 |
| 29 | 4 | 7.50 | 0 |
| 30 | 4 | 8.00 | 0 |
| 31 | 5 | 1.25 | 0 |
| 32 | 5 | 1.88 | 0 |
| 33 | 5 | 2.50 | 0 |
| 34 | 5 | 3.13 | 0 |
| 35 | 5 | 3.75 | 0 |
| 36 | 5 | 4.38 | 0 |
| 37 | 5 | 5.00 | 0 |
| 38 | 5 | 5.63 | 0 |
| 39 | 5 | 6.25 | 0 |
| 40 | 5 | 6.88 | 0 |
| 41 | 5 | 7.50 | 0 |
| 42 | 5 | 8.13 | 0 |
| 43 | 5 | 8.75 | 0 |
| 44 | 5 | 9.38 | 0 |
| 45 | 5 | 10.00 | 0 |
| 46 | 6 | 1.50 | 0 |
| 47 | 6 | 2.25 | 0 |
| 48 | 6 | 3.00 | 0 |
| 49 | 6 | 3.75 | 0 |
| 50 | 6 | 4.50 | 0 |
| 51 | 6 | 5.25 | 0 |
| 52 | 6 | 6.00 | 0 |
| 53 | 6 | 6.75 | 0 |
| 54 | 6 | 7.50 | 0 |
| 55 | 6 | 8.25 | 0 |
| 56 | 6 | 9.00 | 0 |
| 57 | 6 | 9.75 | 0 |
| 58 | 6 | 10.50 | 0 |
| 59 | 6 | 11.25 | 0 |
| 60 | 6 | 12.00 | 0 |
| 61 | 8 | 2.00 | 0 |
| 62 | 8 | 3.00 | 0 |
| 63 | 8 | 4.00 | 0 |
| 64 | 8 | 5.00 | 0 |
| 65 | 8 | 6.00 | 0 |
| 66 | 8 | 7.00 | 0 |
| 67 | 8 | 8.00 | 0 |
| 68 | 8 | 9.00 | 0 |
| 69 | 8 | 10.00 | 0 |
| 70 | 8 | 11.00 | 0 |
| 71 | 8 | 12.00 | 0 |
| 72 | 8 | 13.00 | 0 |
| 73 | 8 | 14.00 | 0 |
| 74 | 8 | 15.00 | 0 |
| 75 | 8 | 16.00 | 0 |
| 76 | 9 | 2.25 | 0 |
| 77 | 9 | 3.38 | 0 |
| 78 | 9 | 4.50 | 0 |
| 79 | 9 | 5.63 | 0 |
| 80 | 9 | 6.75 | 0 |
| 81 | 9 | 7.88 | 0 |
| 82 | 9 | 9.00 | 0 |
| 83 | 9 | 10.13 | 0 |
| 84 | 9 | 11.25 | 0 |
| 85 | 9 | 12.38 | 0 |
| 86 | 9 | 13.50 | 0 |
| 87 | 9 | 14.63 | 0 |
| 88 | 9 | 15.75 | 0 |
| 89 | 9 | 16.88 | 0 |
| 90 | 9 | 18.00 | 0 |
| 91 | 10 | 2.50 | 0 |
| 92 | 10 | 3.75 | 0 |
| 93 | 10 | 5.00 | 0 |
| 94 | 10 | 6.25 | 0 |
| 95 | 10 | 7.50 | 0 |
| 96 | 10 | 8.75 | 0 |
| 97 | 10 | 10.00 | 0 |
| 98 | 10 | 11.25 | 0 |
| 99 | 10 | 12.50 | 0 |
| 100 | 10 | 13.75 | 0 |
| 101 | 10 | 15.00 | 0 |
| 102 | 10 | 16.25 | 0 |
| 103 | 10 | 17.50 | 0 |
| 104 | 10 | 18.75 | 0 |
| 105 | 10 | 20.00 | 0 |
| 106 | 12 | 3.00 | 0 |
| 107 | 12 | 4.50 | 0 |
| 108 | 12 | 6.00 | 0 |
| 109 | 12 | 7.50 | 0 |
| 110 | 12 | 9.00 | 0 |
| 111 | 12 | 10.50 | 0 |
| 112 | 12 | 12.00 | 0 |
| 113 | 12 | 13.50 | 0 |
| 114 | 12 | 15.00 | 0 |
| 115 | 12 | 16.50 | 0 |
| 116 | 12 | 18.00 | 0 |
| 117 | 12 | 19.50 | 0 |
| 118 | 12 | 21.00 | 0 |
| 119 | 12 | 22.50 | 0 |
| 120 | 12 | 24.00 | 0 |
| 121 | 2 | 0.00 | 1 |
| 122 | 3 | 0.00 | 1 |
| 123 | 4 | 0.00 | 2 |
| 124 | 5 | 0.00 | 2 |
| 125 | 7 | 0.00 | 3 |
| 126 | 8 | 0.00 | 3 |
| 127 | 12 | 0.00 | 6 |
| 128 | 12 | 0.00 | 5 |
| 129 | 12 | 0.00 | 4 |
| 130 | 13 | 0.00 | 5 |
| 131 | 13 | 0.00 | 6 |
| 132 | 19 | 0.00 | 8 |
| 133 | 22 | 0.00 | 10 |
| 134 | 23 | 0.00 | 10 |
| 135 | 25 | 0.00 | 9 |
| 136 | 25 | 0.00 | 10 |
| 137 | 26 | 0.00 | 10 |
| 138 | 26 | 0.00 | 12 |
| 139 | 28 | 0.00 | 13 |
| 140 | 30 | 0.00 | 12 |
